# Honey bees rely on associative stimulus strength after training on an olfactory transitive inference task

**DOI:** 10.3389/fpsyg.2024.1529460

**Published:** 2025-01-07

**Authors:** Martin Giurfa, Silvia Lee, Catherine Macri

**Affiliations:** ^1^Sorbonne University, CNRS, INSERM, Institute of Biology Paris Seine, Neurosciences Paris Seine, Paris, France; ^2^Centre de Recherches sur la Cognition Animale, Centre de Biologie Intégrative (CBI), CNRS, UPS, University of Toulouse, Toulouse, France

**Keywords:** transitive inference, stimulus ranking, stimulus associative strength, Pavlovian conditioning, olfactory learning, proboscis extension reflex, honey bee (*Apis mellifera* L.)

## Abstract

Transitive inference, the ability to establish hierarchical relationships between stimuli, is typically tested by training with premise pairs (e.g., A + B–, B + C–, C + D–, D + E–), which establishes a stimulus hierarchy (A > B > C > D > E). When subjects are tested with non-adjacent stimuli (e.g., B vs. D), a preference for B indicates transitive inference, while no preference indicates decisions based on stimulus associative strength, as B and D are equally reinforced. Previous studies with bees and wasps, conducted in an operant context, have shown conflicting results. However, this context allows free movement and the possibility to avoid non-reinforced options, thus reducing the number of non-reinforced trials. To address this, we examined whether honey bees could perform transitive inference using a Pavlovian protocol that fully controls reinforcement. We conditioned bees with five odorants, either forward-or backward-paired with a sucrose solution, across four discrimination tasks. In all experiments, bees showed no preference for B over D, choosing equally between them, regardless of the training schedule. Our results show that bees’ choices were primarily influenced by stimulus associative strength and a recency effect, with greater weight given to the most recent reinforced or non-reinforced stimulus. We discuss these findings in the context of honey bee memory, suggesting that memory constraints may limit cognitive solutions to transitive inference tasks in bees.

## Introduction

Research on animal cognition has shown that some species can rank events based on individual experience (Acuna et al., 2002; Bond et al., 2003). This ability to order events is essential for survival. For instance, in a foraging context, animals can improve efficiency by ranking food items according to factors such as nutritional value, abundance, and other relevant criteria (Davis, 1992). Similarly, in social contexts, hierarchies and dominance relationships often depend on ranking among individuals (Bond et al., 2003; Paz-y-Miño et al., 2004; Grosenick et al., 2007). The ability to establish such relationships between stimuli (A > B; B > C; therefore, A > C) is known as *transitive inference* (Bryant and Trabasso, 1971) and is considered as one of the hallmarks of logical deductive reasoning (Vasconcelos, 2008).

Transitive inference tasks allow researchers to study logical reasoning and knowledge manipulation (Potts, 1974; Woocher et al., 1978; Acuna et al., 2002; Vasconcelos, 2008). It is demonstrated empirically by the ability to infer a relationship (B > D) between non-adjacent items from overlapping premises (A > B, B > C, C > D, D > E) of an underlying series (A > B > C > D > E). A preference for B over D in this context may be attributed to deductive reasoning (von Fersen et al., 1991; Vasconcelos, 2008), where subjects construct and manipulate a unified, linear representation of the implicit hierarchy A > B > C > D > E (Delius and Siemann, 1998; Acuna et al., 2002).

Alternatively, associative theories of transitive inference suggest that animals in this experimental design may respond based on reinforced versus non-reinforced experiences (Werner et al., 1992; Wynne et al., 1992; Siemann and Delius, 1993; Siemann and Delius, 1998; Terrace and McGonigle, 2016). According to this view, animals select stimuli based on associative strength—the number of reinforced versus non-reinforced experiences with each stimulus—rather than relying on deductive reasoning. A critical test to distinguish between these two accounts involves presenting non-adjacent stimuli B and D. If B and D were equally reinforced during training (e.g., A+ vs. B– and B+ vs. C–; C+ vs. D– and D+ vs. E–, where + and – signs indicate the presence and absence of reinforcement, respectively), they would have equivalent associative strengths, as both are equally paired with reinforcement and non-reinforcement. Consequently, subjects guided by associative strength would respond equally to B and D. However, if subjects use a mental representation of the hierarchy learned in training, they should prefer B over D, despite the equal associative strengths.

Beyond humans (Bryant and Trabasso, 1971; Delius and Siemann, 1998), various non-human species have demonstrated the capacity for transitive reasoning. For example, fish (Grosenick et al., 2007), pigeons (von Fersen et al., 1990; von Fersen et al., 1991; Siemann and Delius, 1994; Wynne, 1997), corvids (Bond et al., 2003), pinyon jays (Paz-y-Miño et al., 2004), rats (Davis, 1992; Dusek and Eichenbaum, 1997), squirrel monkeys (McGonigle and Chalmers, 1977; McGonigle and Chalmers, 1992), macaques, (Treichler and Van Tilburg, 1996) and chimpanzees (Gillan, 1981; Boysen et al., 1993) consistently prefer B over D in tests after multiple discrimination training (A+ vs. B–, B+ vs. C–, C+ vs. D–, D+ vs. E–). Transitive inference has been associated with the hippocampus (Dusek and Eichenbaum, 1997; Eichenbaum and Fortin, 2009; Devito et al., 2010), which processes and stores critical relationships among items and events, enabling the flexible use of memories in new situations.

In invertebrates, transitive inference has been studied in an operant context in two insect species—honey bees (Benard and Giurfa, 2004) and wasps (Tibbetts et al., 2019)—with contrasting results. Free-flying honey bees were trained to enter a Y-maze to discriminate between five distinct black-and-white patterns arranged in four overlapping premise pairs, where one stimulus was rewarded with sucrose solution and the other was not (Benard and Giurfa, 2004). This study found no hierarchical ranking of stimuli, as tests with non-adjacent stimuli B and D showed no preference, indicating that choices were guided by the associative strength of each stimulus (Benard and Giurfa, 2004). *Polistes* wasps were trained with five colors arranged in four overlapping premise pairs displayed on opposite walls of a rectangular box, where one color was paired with electric shock and the other was not (Tibbetts et al., 2019). After training, unlike bees, wasps preferred B over D when tested with these non-adjacent stimuli, indicating a hierarchy of colors based on transitive inference (Tibbetts et al., 2019).

Both studies relied on operant training, raising the issue of reinforcement control, as insects in these setups could move freely. Consequently, reinforcement outcomes depended on the insect’s choices and actions. With free movement and choice, the animals may quickly learn to avoid non-reinforced alternatives, resulting in fewer non-reinforced experiences than initially planned by the experimenter. This highlights the need for precise control over the reinforcement history of each stimulus to determine whether the animal’s choices are influenced by associative factors or transitive inferences. In the case of honey bees, addressing the transitive inference problem with full control over reinforcement history is achievable using a Pavlovian conditioning protocol called olfactory conditioning of the proboscis extension response (PER) (Bitterman et al., 1983; Menzel, 1999; Giurfa, 2007; Giurfa and Sandoz, 2012). In this protocol, restrained honey bees learn to associate olfactory stimuli with a sucrose solution reward. When the antennae of a hungry bee are touched with sucrose solution, it reflexively extends its proboscis to consume the sucrose. While odors alone do not trigger this reflex in naive bees, forward pairing of an odor with sucrose creates an association, allowing the odor to elicit a PER in subsequent tests (Bitterman et al., 1983). In this protocol, the odor acts as the conditioned stimulus (CS), while the sucrose solution serves as the reinforcing unconditioned stimulus (US). Since reinforcement delivery is entirely controlled by the experimenter, the bees’ responses do not influence the learning of the odor-sucrose association (Bitterman et al., 1983). In differential conditioning, where bees must learn to discriminate between a rewarded and a non-rewarded odorant, the protocol allows for the delivery of both reinforced trials (CS+ trials) and non-reinforced trials (CS– trials), in which no reward is provided. An even more effective approach for CS– trials involves presenting the unconditioned stimulus (US) before the conditioned stimulus (CS) in a backward pairing. This method induces inhibitory learning of the CS– (Hellstern et al., 1998), leading to improved discrimination (Schleyer et al., 2018).

The PER protocol has been widely used to study various learning and discrimination tasks in the olfactory domain (see review in Giurfa and Sandoz, 2012). However, no attempts have yet been made to investigate transitive inference, despite the feasibility of conditioning premise pairs using the PER paradigm. This approach allows precise control over the number of excitatory (+) and inhibitory (−) experiences the bees have with each stimulus in the series—a level of control that is difficult to achieve in operant conditioning setups.

We trained honey bees using differential olfactory conditioning of the proboscis extension reflex (PER), in which one odor was rewarded with a sucrose solution (CS+ trials), while the other was backward paired with the sucrose solution (–CS trials; the minus sign was inverted to account henceforth for the backward US delivery). Bees were conditioned with a sequence of four premise pairs of odorants arranged into a defined hierarchy (A > B > C > D > E or A < B < C < D < E). Our aim was to determine whether honey bees could form transitive inferences or if they relied on the associative strength of the stimuli experienced under these conditions, and to explore the mechanisms underlying their responses.

## Materials and methods

### Subjects

Honey bee foragers (*Apis mellifera*) were captured as they landed on a feeder containing a 30% sucrose solution (w/w) to which they had been previously trained. The experiments were conducted during late spring and summer, when training to such a feeder is feasible. Each captured bee was placed in a small glass vial and immobilized by cooling in a freezer at −6°C for 3 to 4 min. The bees were then harnessed in small tubes, with the head protruding, allowing only the movement of the antennae and mouthparts, including the proboscis. Afterward, each bee was fed 4 μL of the 30% sucrose solution and left undisturbed in a dark box with moist filter paper for 2 h. Ten minutes before each experiment, bees were tested for intact proboscis extension reflex (PER) by lightly touching their antennae with a toothpick dipped in a 30% sucrose solution (w/w). Extension of the proboscis beyond an imaginary line between the open mandibles was counted as PER (the unconditioned response). Bees that did not show the reflex (<5%) were excluded from the experiments.

### Unconditioned and conditioned stimuli

The unconditioned stimulus (US) was always a 30% sucrose solution (w/w). The conditioned stimuli (CSs) were the odorants 1-Hexanol, 2-Hexanone, Heptanal, 2-Nonanol and Eugenol (all obtained from Sigma-Aldrich, Deisenhofen, Germany), which are well differentiated in olfactory PER conditioning experiments. The choice of these odorants was based on a generalization matrix that includes four of the five odorants used (1-Hexanol, 2-Hexanone, Heptanal, and 2-Nonanol), showing low cross-generalization (Guerrieri et al., 2005). Additionally, we had preliminary data for the missing comparisons involving Eugenol, indicating low generalization.

Four microliters of each odorant were applied to a fresh strip of filter paper, which was then placed inside a 20 mL plastic syringe. During each trial, a scented airflow was directed toward the bees’ antennae by gently pressing the syringe from a distance of approximately 5 cm. An exhaust system was positioned behind the bees to remove odor-laden air.

In all three experiments, within each group, and for each bee, a specific odorant was assigned to categories A, B, C, D, and E. The odorant sequence was then shifted for each subsequent bee, ensuring that each odorant was evenly distributed across the categories.

### Conditioning

Differential conditioning was used in all experiments, whereby animals learn to respond to a reinforced odorant while inhibiting responses to a non-reinforced odorant. In preliminary experiments using the same odorants, we observed a high rate of generalization between reinforced and non-reinforced odorants. To mitigate this effect, and to achieve a full control of reinforcement history, we implemented differential conditioning in which reinforced trials (CS+ trials) involved forward pairing of the CS and the US, while non-reinforced trials (–CS trials) involved backward pairing, where the US was presented before the CS to reduce responses to the –CS (Hellstern et al., 1998). The use of the backward pairing, which induces inhibitory learning of the –CS (i.e., learning that the CS is not followed by the US; Hellstern et al., 1998), was precisely aimed at reducing odor generalization.

Each trial began by placing the subject 15 cm in front of the exhaust system, where it acclimated for 15 s. In CS+ trials, the CS was presented before the US (forward conditioning): the CS began at the 15-s mark, followed by the US 2 s later. Both the CS and the US were presented for 4 s. The US was delivered by lightly touching the antennae with a toothpick dipped in sucrose solution, allowing the bee to feed for 2 s after proboscis extension. This created a 2-s interstimulus interval with a 2-s overlap between the CS and the US. In –CS trials, the US was delivered first (backward conditioning), starting at the 15-s mark, followed by the CS 2 s later. Both stimuli lasted for 4 s, with a 2-s interstimulus interval and overlap. Each trial, regardless of pairing type, concluded at the 30-s mark. After each trial, bees were returned to their resting positions. A total of 12 bees were trained per experimental run, with a 6-min interval between trials.

### Testing

At the end of each conditioning phase, an intermediate retention test was conducted to verify whether the bees had learned the discrimination. These tests determined if the bees had learned not only to respond to the CS+ but also to refrain from responding to the –CS, which could not be evaluated though the learning curves, as responses during the –CS conditioning trials were not measurable (see above). During these tests, bees were presented with the two odors from the just-completed phase without any reward. After completing all five conditioning phases, the bees were additionally subjected to five final tests where they were tested on the five trained odors (A, B, C, D, E) in a random sequence without reinforcement. During intermediate and final tests, the duration of odorant delivery, and the interstimulus interval (inter-test interval) were the same as during conditioning trials.

### Response measurement

For CS+ trials, we recorded whether or not a bee extended its proboscis after the onset of the CS and before US delivery, which was counted as the conditioned response. Multiple responses during a single CS were counted as one PER. For –CS trials, no response to the CS could be recorded, as bees always responded to the US presented before the CS. In this case, bees continued extending the proboscis during the overlapping –CS but this cannot be counted as a CS response. Thus, acquisition curves reflect only the variation in responses to the CS+ across trials. At the end of each experiment, all animals were retested for the proboscis extension reflex to the US; bees that did not respond (<5%) were excluded from the analyses.

### Experiments

#### Experiment 1

Two groups of bees (*Group I* and *Group II*) were trained on a sequence of four odor discriminations involving five different odorants. Each discrimination phase employed differential conditioning, with the conditioned stimulus (CS+) and the non-conditioned stimulus (–CS) each presented six times, totaling 48 trials (Table 1).

**Table 1:** Experimental groups, sample size (*n*) and training sequences of Experiments 1–3.

Experiment	Group	*n*	Conditioning phases
**Experiment 1** (6 CS+ and 6 –CS trials per training phase, 4 training phases, i.e., 48 trials)	*Group I*	43	A+ vs. –B, B+ vs. –C, C+ vs. –D, D+ vs. –E
	*Group II*	44	–A vs. B+, –B vs. C+, –C vs. D+, –D vs. E+
**Experiment 2** (6 CS+ and 6 –CS trials per training phase, 4 training phases, i.e., 48 trials)	*Group III*	40	C+ vs. –D, D+ vs. –E, A+ vs. –B, B+ vs. –C
	*Group IV*	41	–C vs. D+, –D vs. E+, −A vs. B+,–B vs. C+
**Experiment 3** (2 CS+ and 2 –CS trials per training phase, 12 training phases, i.e., 48 trials)	*Group V*	43	C+ vs. –D, A+ vs. –B, D+ vs. –E, B+ vs. –C, D+ vs. –E, C+ vs. –D, B+ vs. –C, A+ vs. –B, B+ vs. –C, D+ vs. –E, A+ vs. –B, C+ vs. –D

A, B, C, D, E indicate different odorants; + and – indicate forward and backward pairing of sucrose solution, respectively.

*Group I* was trained with the sequence A+ vs. –B, B+ vs. –C, C+ vs. –D, and D+ vs. –E, while *Group II* received the reverse sequence: –A vs. B+, –B vs. C+, –C vs. D+, and –D vs. E+. These correspond to the hierarchies A > B > C > D > E for *Group I* and A < B < C < D < E for *Group II.*

As mentioned above, at the end of each conditioning phase, a retention test was conducted to verify whether the bees had learned the discrimination. During each test, bees were presented with the two odors from the just-completed phase without any reward. To account for potential extinction effects, each odor pair was retrained immediately following the retention test, with two presentations of the CS+ and two of the –CS in random order. These refreshment trials were not included in the analyses.

In addition to recording responses to the CS+ and the –CS, a differentiation index (*Δ*) was calculated for each test as the difference between responses (R) to the CS+ and to the –CS (i.e., Δ = R_CS+_ − R_-CS_). This index enabled comparison of discrimination performance at the end of each conditioning phase.

Upon completion of all five conditioning phases, bees were presented with the five trained odors (A, B, C, D, E) in a random sequence without any reward. If the bees had formed transitive inferences from conditioning, *Group I* bees should prefer B over D, while *Group II* bees should prefer D over B, despite these two odors never being presented together during training. If no preference between B and D was observed, it would suggest that the bees relied on the associative strength of the stimuli, which was equal for both B and D.

#### Experiment 2

Two groups of bees (*Group III* and *Group IV*) were trained using a sequence of four odor discriminations involving five different odors, following the same experimental protocol as in Experiment 1. Each discrimination phase involved differential conditioning, where each CS+ and –CS was presented six times, resulting in a total of 48 trials. The key difference in this experiment was the arrangement of the conditioning pairs. Instead of following a sequence aligned with the stimulus hierarchy, we positioned the unambiguous stimuli (e.g., A+ and E–) in the middle of the training schedule rather than at the ends (Table 1).

*Group III* was trained with the following sequence: C+ vs. –D, D+ vs. –E, A+ vs. –B, and B+ vs. –C. *Group IV*, on the other hand, was trained with the reverse sequence: –C vs. D+, –D vs. E+, −A vs. B+, and –B vs. C+. Despite these different conditioning sequences, the stimulus hierarchy established in *Group III* mirrored that of *Group I* (i.e., A > B > C > D > E), while the hierarchy in *Group IV* matched that of *Group II* (i.e., A < B < C < D < E).

At the end of each conditioning phase, a retention test was conducted. During this test, the two odors from the most recent training phase were presented in a random sequence without reward. To address potential extinction effects, the same odor pair was then retrained with two CS+ and two –CS presentations in random order. These tests assessed whether the bees learned not only to respond to the CS+ but also to inhibit responses to the –CS. After completing all five conditioning phases, the bees were exposed to the five trained odors (A, B, C, D, E) in a random sequence, again without reward.

#### Experiment 3

The previous two experiments used sequential training, in which each discrimination was trained during a fixed number of consecutive trials. However, a substantial amount of research on transitive inferences in animals has used intermixed training, where all discriminations are trained concurrently (see Vasconcelos, 2008 for review). Therefore, we designed Experiment 3 to resemble an intermixed training approach. Each of the original conditioning phases used in Experiments 1 and 2, which included 12 trials (6 CS+ and 6 –CS), was replaced with three shorter phases, each consisting of four trials (2 CS+ and 2 –CS), presented pseudorandomly. For example, instead of an A+ vs. –B phase with 6 A+ and 6 –B trials, the bees experienced three A+ vs. –B phases, each with 2 A+ and 2 –B trials. This adjustment maintained a total of 48 trials (Table 1).

A single group of bees (*Group V*) was trained with the following sequence: C+ vs. –D, A+ vs. –B, D+ vs. –E, B+ vs. –C, D+ vs. –E, C+ vs. –D, B+ vs. –C, A+ vs. –B, B+ vs. –C, D+ vs. –E, A+ vs. –B, C+ vs. –D. This design ensured that no odor pair appeared in a fixed sequential order, thereby eliminating potential biases from order effects. Despite the varied sequence of conditioning pairs, the stimulus hierarchy established in *Group V* was identical to that of *Group I* (Experiment 1) and *Group III* (Experiment 2), i.e., A > B > C > D > E. Intermediate retention tests were not conducted in this experiment to avoid potential extinction effects from repeated non-rewarded trials. At the end of training, the bees were exposed to the five trained odors (A, B, C, D, E) in a random sequence, again without reward.

### Statistical analysis

We calculated the percentage of conditioned proboscis extension responses (%PER) during the CS+ trials in the acquisition phases. No conditioned responses could be recorded during the –CS trials, as delivering sucrose first in backward trials already elicited a response before CS presentation. During the tests, the percentage of PER was recorded for all odorants under extinction conditions.

Learning performance was analyzed using a GLMER for binomial family, where individual identity was treated as a random factor. Trial (six levels, from one to six), Phase (four levels: A+, B+, C+, D+ for *Groups I*, *III* and *V*, or B+, C+, D+, and E+ for *Groups II* and *IV*), and their interaction were included as main effects. A comprehensive statistical report for each group (I to V) during the conditioning phase is available in the Supplementary materials. For each experiment, we selected the best model based on an information-theoretic approach and *Δ*AIC (Akaike Information Criterion) comparisons using the MuMIN package. When the main effect of ‘Phase’ was significant, we conducted a Tukey *post hoc* analysis with the lsmeans package to compare all possible pairs of phases. *Z*-values and *p*-values are reported throughout.

Performance in the intermediate retention tests conducted after each conditioning phase was analyzed using a McNemar test. Differentiation indices (Δ) in these tests were compared with a Friedman test, followed by a *post-hoc* analysis using a pairwise signed-ranks test. Performance in the final test was analyzed with a Cochran test, allowing comparison of multiple proportions from binary data in a repeated-measure design. *Post-hoc* multiple comparisons with Bonferroni alpha adjustment (*α* = 0.005) were performed by calculating the minimum required difference (MRD) for any pair of proportions to be considered significantly different (Sheskin, 2011).

Statistical analyses were conducted using the R software (version 4.3.2, R project) and Statistica (version 13.3, Tibco Software).

## Results

### Experiment 1

In this experiment, we trained two groups of bees using a sequence of four odor discriminations involving five distinct odors. *Group I* (*n* = 43 bees) underwent the following sequence of pairings: A+ vs. –B, B+ vs. –C, C+ vs. –D, and D+ vs. –E. Conversely, *Group II* (*n* = 44 bees) was trained using the reverse sequence of pairings: –A vs. B+, –B vs. C+, –C vs. D+, and –D vs. E+. These pairings represent stimulus hierarchies of A > B > C > D > E and A < B < C < D < E, respectively.

**Figure 1:**
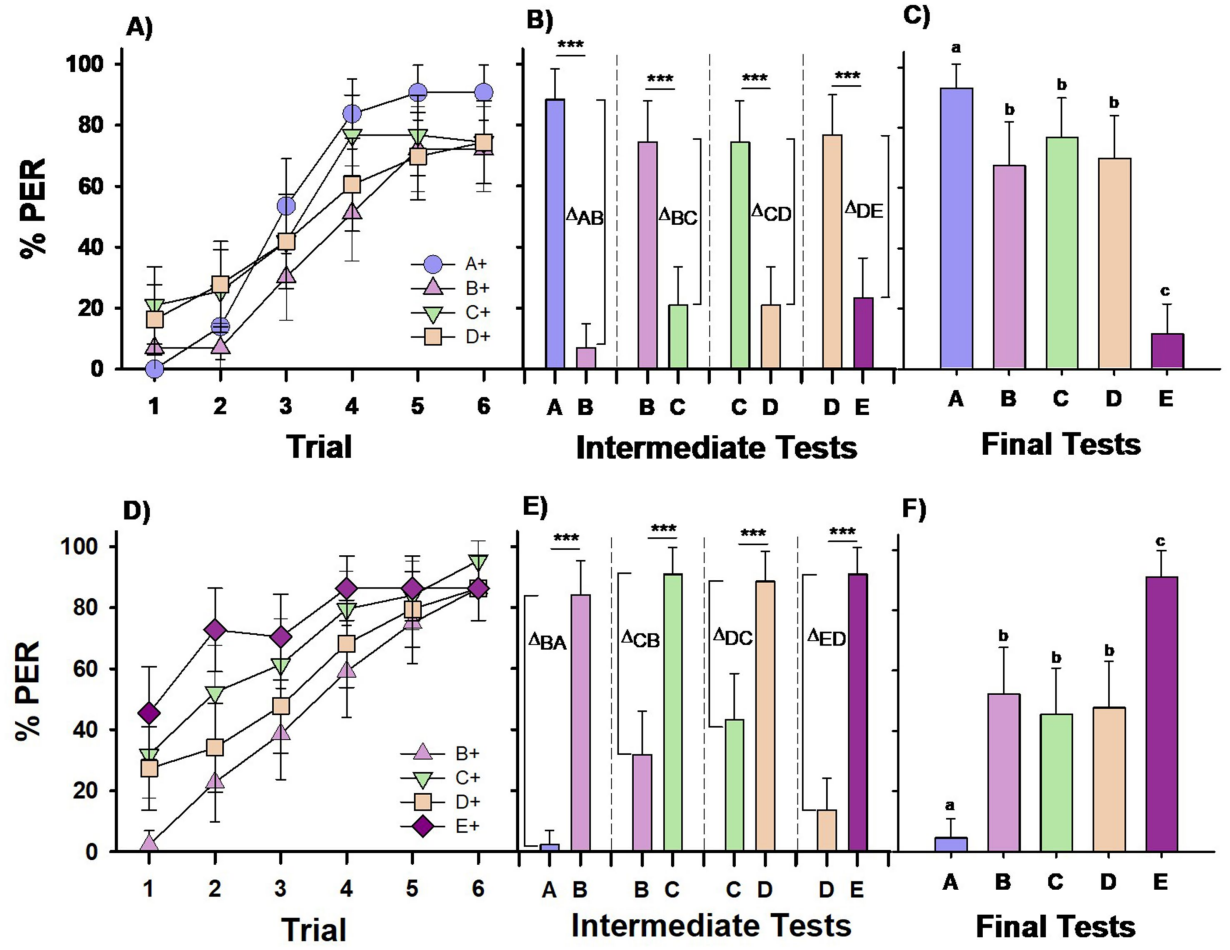
Experiment 1. *Group I* **(A–C)** and *Group II* **(D–F)**: Two groups of bees were differentially trained with four premise pairs across four successive phases. *Group I* (*n* = 43) was conditioned to the sequence A+ vs. –B, B+ vs. –C, C+ vs. –D, D+ vs. –E (upper row). *Group II* (*n* = 44) was conditioned to the reversed sequence –A vs. B+, –B vs. C+, –C vs. D+, –D vs. E+ (lower row); ‘+’ and ‘–’ indicate forward and backward pairing with sucrose solution, respectively. Each phase included 6 CS+ (forward-paired) and 6 –CS (backward-paired) trials. **(A,D)** Percentage of PER (proboscis extension response) across trials for each of the four different CS+ stimuli in the training sequence. **(B,E)** Percentage of PER in intermediate tests conducted at the end of each training phase, where the stimuli from the completed phase were presented without reward. *Δ* values represent the differentiation indices calculated on the basis of responses (*R*) to the trained stimuli (Δ = R_CS+_ − R_-CS_). ***p < 0.00001. **(C,F)** Percentage of PER in the final test at the end of the training sequence, where all five test stimuli were presented without reward. Different letters on top of bars indicate significant differences after a Cochran test and *post-hoc* multiple comparisons with Bonferroni alpha adjustment (*α* = 0.005).

Bees in *Group I* learned to respond to the CS+ in each discrimination phase (Figure 1A). No significant difference was observed between phases when referring performance to that in the initial phase (A+ vs. –B) (GLMER; phase effect; A+ vs. B+: *z* = 0.9, *p* = 0.37; A+ vs. C+: *z* = 0.99, *p* = 0.32, A+ vs. D+: *z* = 0.97, *p* = 0.33).

Intermediate retention tests, conducted at the end of each discrimination phase, revealed that the bees consistently learned to respond to the previously reinforced odor (CS+) and inhibit responses to the non-reinforced odor (–CS) (Figure 1B; McNemar test: *p* < 0.00001 for all four tests). A comparison of the differentiation index across these tests indicated a significant difference in the level of differentiation achieved (Friedman test: χ^2^ = 10.62, df: 3, *p* = 0.014). Post-hoc comparisons showed significantly better differentiation in the first test, A vs. B (*p* < 0.05 for A vs. B compared to tests C vs. B, C vs. D and D vs. E), while differentiation levels across the remaining three tests did not significantly differ (*p* = 0.98 for all comparisons).

During the final test, in which all five odors were presented, the bees’ responses varied significantly depending on the odor (Figure 1C; Cochran test: Q = 63.75, df = 4, *p* < 0.00001). Bees responded significantly more to the top stimulus in the hierarchy, which was always rewarded (stimulus A; MRD, *p* < 0.005 for all pairwise comparisons of A vs. the other four stimuli) and significantly less to the bottom stimulus, which was never rewarded (stimulus E; *p* < 0.005 for all pairwise comparisons of E vs. the other four stimuli). Responses to stimuli B, C, and D were similar (NS) and intermediate between A and E. Therefore, bees in *Group I* did not show a preference for B over D, and their responses did not indicate the formation of transitive inferences. The similarity in response rates to B and D, along with distinct responses to A and E, suggests that the bees’ behavior was driven by the associative strength of each stimulus in this task.

Bees of *Group II,* trained with a reversed sequence of pairings compared to *Group I,* learned to respond to the CS+ in each discrimination phase (Figure 1D). Unlike *Group I,* the learning curves for bees in *Group II* varied significantly across conditioning phases, with the performance during the 1st phase (−A vs. B+) taken as a reference (GLMER; phase effect; B+ vs. C+: *z* = 5.25, *p* < 0.0001; B+ vs. D+: *z* = 2.59, *p* = 0.009, B+ vs. E+: *z* = 7.08, *p* < 0.0001). This difference resulted from the initial response to each newly introduced stimulus, which in each phase was a CS+. This effect was absent in *Group I,* where the newly introduced stimulus was consistently a –CS, making it impossible to observe a response to it.

Intermediate retention tests conducted at the end of each discrimination phase showed that bees consistently learned to distinguish the CS+ from the –CS (Figure 1E; McNemar test: *p* < 0.00001 for all four tests). Significant differences in differentiation (*Δ*) were observed across the four tests (Figure 1E; χ^2^ = 16.13; df = 3; *p* < 0.005). Differentiation was stronger in the first test (B vs. A) than in tests C vs. B and D vs. C (*p* < 0.05 for both), likely due to A’s consistent inhibitory role, whereas B, C, and D could act as either excitatory or inhibitory stimuli. Differentiation levels in the second and third tests (C vs. B and D vs. C) did not differ significantly (*p* = 0.20). In the fourth test (D vs. E), the level of differentiation was intermediate; it did not differ significantly from the levels in tests A vs. B and C vs. B (*p* = 0.64 and *p* = 0.07, respectively) but was significantly higher than that in test D vs. C (*p* < 0.005).

In the final test, where all five odors were presented, bees’ responses varied significantly depending on the odor (Figure 1F; *Q* = 61.08, df = 4, *p* < 0.00001). Bees responded significantly more to the stimulus at the top of the hierarchy, which was always rewarded (stimulus E; MRD, *p* < 0.005 for all pairwise comparisons of E vs. the other four stimuli), and significantly less to the bottom stimulus, which was never rewarded (stimulus A; *p* < 0.005 for all pairwise comparisons of A vs. the other four stimuli). Responses to stimuli B, C, and D were similar (NS) and intermediate between E and A. Thus, bees in *Group II* did not show a preference for D over B, indicating that they had not formed transitive inferences. The bees’ responses appeared to be guided by the associative strength of each stimulus, as demonstrated by their clear preference for E and lower response to A.

### Experiment 2

In this experiment, we trained two groups of bees on a sequence of four odor discriminations involving five different odors, similar to Experiment 1. However, unlike Experiment 1, the training sequence was arranged so that the unambiguous stimuli (e.g., A+ and E–) appeared in the middle of the sequence rather than at the ends.

One group (*Group III*; *n* = 40 bees) was trained with the following sequence: C+ vs. –D, D+ vs. –E, A+ vs. –B, and B+ vs. –C. The other group (*Group IV*; *n* = 41 bees) was trained with the reverse sequence: –C vs. D+, –D vs. E+, −A vs. B+, and –B vs. C+. The stimulus hierarchy for *Group III* was identical to that of *Group I* (i.e., A > B > C > D > E), while the hierarchy in *Group IV* matched that of *Group II* (i.e., A < B < C < D < E).

**Figure 2:**
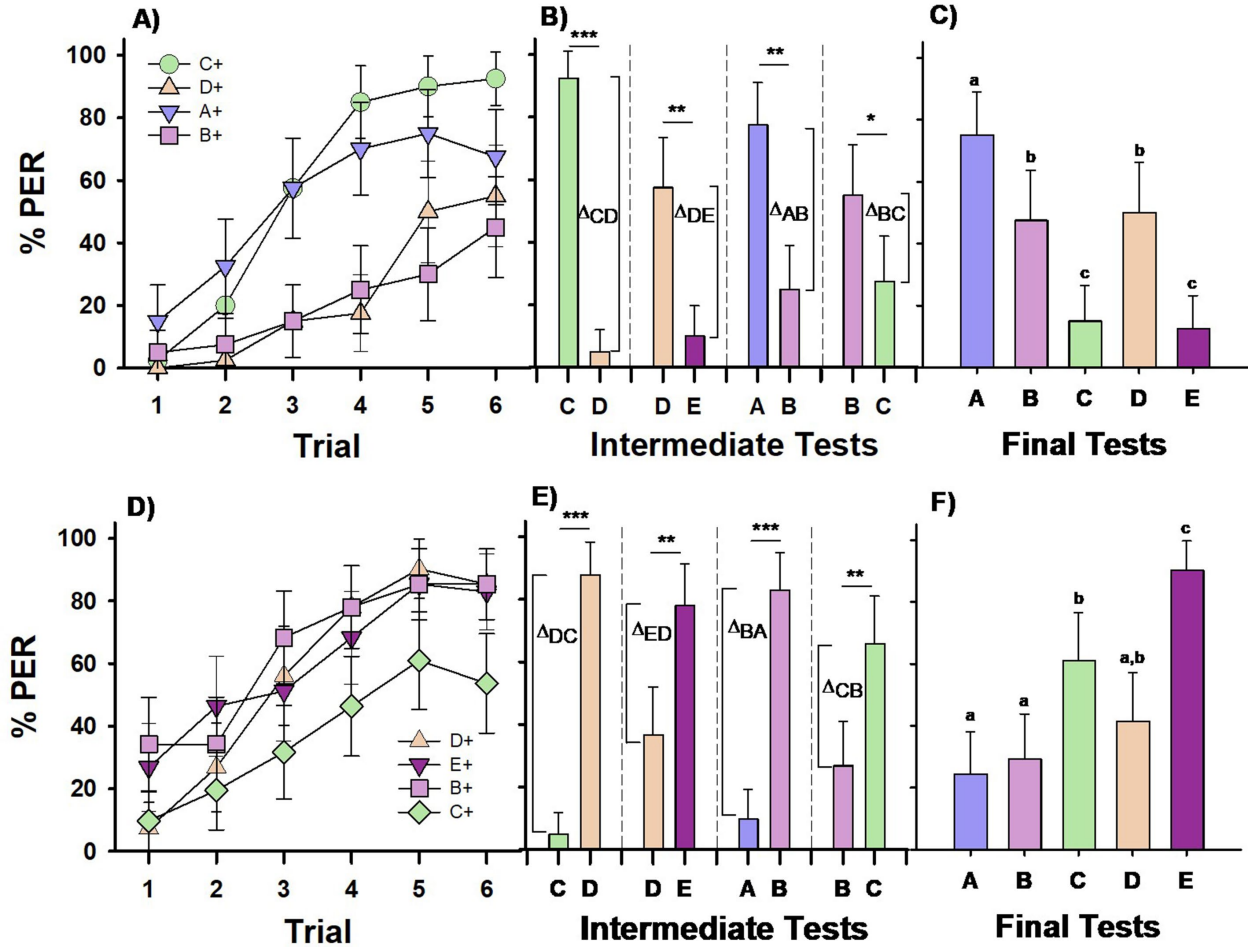
Experiment 2. *Group III* **(A–C)** and *Group IV* **(D–F)**: two groups of bees were differentially trained with four premise pairs across four successive phases. *Group III* (*n* = 40) was conditioned to the sequence C+ vs. –D, D+ vs. –E, A+ vs. –B, B+ vs. –C, (upper row). *Group IV* (*n* = 41) was conditioned to the reversed sequence –C vs. D+, –D vs. E+, −A vs. B+, –B vs. C+, (lower row). Each phase included 6 CS+ (forward-paired) and 6 –CS (backward-paired) trials. **(A,D)** Percentage of PER (proboscis extension response) across trials for each of the four different CS+ stimuli in the training sequence. **(B,E)** Percentage of PER in intermediate tests conducted at the end of each training phase, with stimuli from the completed phase presented without reward. Δ values represent the differentiation indices calculated on the basis of responses (R) to the trained stimuli (Δ = R_CS+_ − R_-CS_). ***p < 0.00001; **p < 0.001; *p < 0.05. **(C,F)** Percentage of PER in the final test at the end of the training sequence, with all five test stimuli presented without reward. Different letters on top of bars indicate significant differences after a Cochran test and *post-hoc* multiple comparisons with Bonferroni alpha adjustment (α = 0.005).

Bees of *Group III* successfully learned all four discriminations (Figure 2A) but their performance varied across phases, using the initial phase (C+ vs. D–) as a reference (GLMER; phase effect; C+ vs. D+: *z* = −8.83, *p* < 0.0001; C+ vs. A+: *z* = −1.33, *p* = 0.18, C+ vs. B+: *z* = −9.28, *p* < 0.0001). Bees performed best in the first (C+ vs. –D) and third (A+ vs. –B) discriminations. There was no significant difference in performance between the first and third phases (C+ vs. A+: *z* = 1.33, *p* = 0.54) or between the second (D+ vs. –E) and the fourth (B+ vs. –C) phases (D+ vs. B+: *z* = −0.65, *p* = 0.91). However, notable differences were found across these groups (C+ vs. D+: *z* = 8. 83, *p* < 0.0001; C+ vs. B+: *z* = 9.28, *p* < 0.0001; D+ vs. A+: *z* = 7.86, *p* < 0.0001; A+ vs. B+: *z* = 8.34, *p* < 0.0001). The improved performance in the first and third phases likely stems from the introduction of a new rewarded, unambiguous stimulus in each case: C+ and A+, respectively.

Retention tests conducted at the end of each discrimination phase confirmed that the bees learned to differentiate the CS+ from the –CS in all cases (Figure 2B; McNemar test: *p* < 0.05 for all four tests). However, significant differences in the level of differentiation (*Δ*) were observed across the four tests (Figure 2B; χ^2^ = 21.70; df = 3; *p* < 0.0001). Differentiation was significantly stronger in the first test (C vs. D; *p* < 0.01 compared to the other three tests), while differentiation in the remaining tests (D vs. E, A vs. B, and B vs. C) did not differ significantly.

The results of the final test (Figure 2C) showed a response pattern similar to, but distinct from, that of *Group I* of Experiment 1. Bees’ responses varied significantly according to the tested odor (*Q* = 97.47, df = 4, *p* < 0.00001). Bees responded significantly more to the stimulus that was always rewarded and was at the top of the hierarchy, even though it was not presented first in the training sequence (stimulus A; MRD, *p* < 0.005 for all pairwise comparisons of A vs. the other four stimuli). They responded less to stimulus E, which was at the bottom of the hierarchy and was never rewarded, and to stimulus C, the final non-rewarded stimulus (*p* < 0.005 for the pairwise comparisons of C vs. A, B, and D, and E vs. A, B, and D). The comparison between C and E was not significant. The decreased responses to C can, therefore, be attributed to a recency effect, which places greater emphasis on the most recent events (Baddeley and Hitch, 1993). Responses to B and D were similar (NS), indicating that Group III bees showed no preference for B over D. Thus, their responses did not support transitive inference formation but were instead guided by the associative strength of each stimulus. This conclusion is supported by their clear preference for A, which was always rewarded, and reduced responses to E, which was consistently unrewarded, regardless of presentation order during training. Additionally, a recency effect was evident in the reduced response to C, the last non-rewarded stimulus.

Bees of *Group IV* showed also differences in acquiring the four discrimination tasks (Figure 2D). This difference was introduced by the last discrimination phase as revealed by a GLMER analysis using performance in the first phase (–C vs. D+) as a reference (D+ vs. E+: *z* = 0.77, *p* = 0.44; D+ vs. B+: *z* = 1.88, *p* = 0.06, D+ vs. C+: *z* = −5.28, *p* < 0.0001). *Post hoc* analyses confirmed that performance in the last discrimination phase also differed significantly from that in the second and third phases (C+ vs. E+: *z* = −5.98, *p* < 0.0001; C+ vs. B+: *z* = 6.95, *p* < 0.0001). This effect likely arose because C+ had undergone backward conditioning during the initial phase (–C vs. D+), whereas the other CS+ stimuli (D+, E+, B+) had not been subjected to this kind of conditioning before being presented as CS+ (see Table 1). Consequently, C+ reached a lower response level by the end of conditioning than the other three stimuli (D+, E+, B+), which did not significantly differ from each other (see above for D+ vs. E+ and D+ vs. B+; E+ vs. B+: *z* = 1.12, *p* = 0.68).

Retention tests conducted at the end of each discrimination phase showed that *Group IV* bees consistently learned to discriminate the CS+ from the –CS (Figure 2E; χ^2^ = 21.70, df = 3, *p* < 0.001 for all four tests). There was a significant difference in the level of differentiation (*Δ*) across the four tests (Figure 2E; χ^2^ = 63.85, df = 3, *p* < 0.0001). Differentiation was significantly higher in the first and third tests (C vs. D and A vs. B, respectively; *p* < 0.001), while differentiation in the second test (D vs. E) was significantly greater than in the fourth test (B vs. C; *p* < 0.001).

The final test results (Figure 2F) revealed that bees’ responses varied significantly depending on the odor presented (*Q* = 127.75, df = 4, *p* < 0.00001). Bees responded significantly more to stimulus E, which was at the top of the hierarchy as it was consistently rewarded (stimulus E; MRD, *p* < 0.005 for all pairwise comparisons of E vs. the other four stimuli). Responses to B and D were similar (NS), while the response to C was intermediate between those to E and A. The increased response to C may reflect a recency effect, as it was the last rewarded stimulus in the training sequence. Consequently, *Group IV* bees did not show a preference for D over B, and their performance did not support the formation of transitive inferences. Their responses were driven by the associative strength of the stimuli and influenced by a recency effect.

### Experiment 3

The previous experiments indicated that, in addition to relying on the associative strength of stimuli, bees also placed more weight on the last discrimination, demonstrating that their choices were influenced by a recency effect. In this final experiment, we aimed to eliminate the effects caused by the appearance of an odor pair in a specific and constant sequential order. To achieve this, we trained a single group of bees (*Group V*; n = 43) with the following sequence: C+ vs. –D, A+ vs. –B, D+ vs. –E, B+ vs. –C, D+ vs. –E, C+ vs. –D, B+ vs. –C, A+ vs. –B, B+ vs. –C, D+ vs. –E, A+ vs. –B, C+ vs. –D. The stimulus hierarchy for this group was identical to that of *Group I* (Experiment 1) and *Group III* (Experiment 2), i.e., A > B > C > D > E.

**Figure 3:**
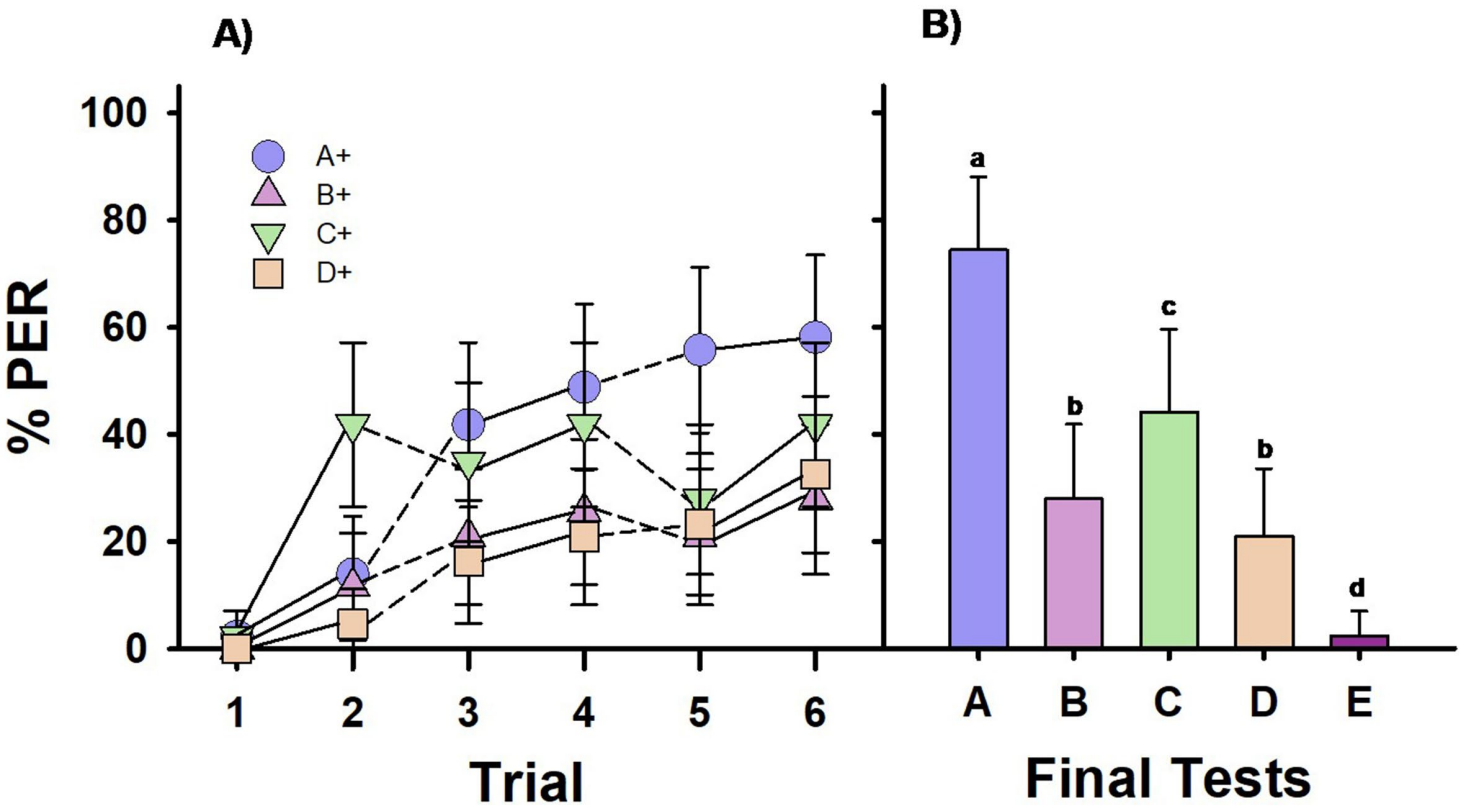
Experiment 3. Group V: One group of bees (*Group V*; *n* = 43 bees) was differentially trained with four premise pairs along 12 pseudo-randomized successive phases: C+ vs. –D, A+ vs. –B, D+ vs. –E, B+ vs. –C, D+ vs. –E, C+ vs. –D, B+ vs. –C, A+ vs. –B, B+ vs. –C, D+ vs. –E, A+ vs. –B, C+ vs. –D. Each phase consisted of 2 CS+ (forward-paired) and 2 –CS (backward-paired) presentations. **(A)** Percentage of PER (proboscis extension response) across trials for each of the four different CS+ stimuli in the training sequence. Solid lines connect consecutive trials. Dashed lines connect non-consecutive trials. **(B)** Percentage of PER in the final test at the end of the training sequence, where all five test stimuli were presented without reward. Different letters on top of bars indicate significant differences after a Cochran test and *post-hoc* multiple comparisons with Bonferroni alpha adjustment (α = 0.005).

Figure 3A shows the performance of *Group V* during training. The learning curves display the performance across the four discriminations (i.e., each curve shows the bees’ responses across the three brief phases of a given discrimination, totaling six CS+ presentations per CS+). The acquisition levels reached at the end of conditioning were lower than those observed in Experiments 1 and 2, likely due to the challenges associated with interspersing various short conditioning phases. Although bees successfully learned the four discrimination tasks, there were differences across learning curves. Two categories of performance emerged: bees were better at learning discriminations C+ vs. –D and A+ vs. –B than discriminations D+ vs. –E and B+ vs. –C. GLMER analysis confirmed this difference, showing no significant differences between learning curves for C+ vs. –D and A+ vs. –B (C+ vs. A+: *z* = 1.47, *p* = 0.14) or between learning curves for D+ vs. –E and B+ vs. –C (D+ vs. B+: *z* = 0.54, *p* = 0.98), but revealing significant differences when comparing these two categories (C+ vs. D+: z = –4.77, *p* < 0.0001; C+ vs. B+: *z* = 4.29, *p* = 0.0001; A+ vs. D+: *z* = 6.07, *p* < 0.0001; A+ vs. B+: *z* = 5.61, *p* < 0.0001). The higher learning rate for A+ vs. –B may be due to A+ being the only stimulus that was never backwardly conditioned. In the case of C+ vs. –D, improved learning may have resulted from its rewarding position at both the beginning and end of the training sequence (Table 1). Conversely, the lower learning rate B+ vs. –C and D+ vs. –E may be attributed to the fact that their first presentations in the training sequence were backwardly associated with sucrose (–D and –B; Table 1).

The results of the final test (Figure 3B) reveal that *Group V*’s responses varied significantly based on the stimulus tested (*Q* = 55.63, df = 4, *p* < 0.00001). Despite the pseudo-randomized conditioning phases, bees responded significantly more to the highest-ranked stimulus (stimulus A; *p* < 0.005 for all pairwise comparisons of A vs. the other four stimuli) and significantly less to the lowest-ranked stimulus (stimulus E; *p* < 0.005 for all pairwise comparisons of E vs. the other four stimuli). The responses to B and D were similar (NS), while responses to C were significantly higher (*p* < 0.005). Therefore, *Group V* bees did not show a preference for B over D, and their performance did not indicate transitive-inference formation. The comparable responses to B and D suggest that the associative strength of the stimuli was the main factor influencing bees’ choices. The significant increase in responses to C can again be attributed to a recency effect, as the final conditioning pair in the sequence was C+ vs. –D.

## Discussion

This study investigated whether honey bees can form transitive inferences when trained with a series of premise odor pairs (e.g., A+ vs. –B, B+ vs. –C, C+ vs. –D, D+ vs. –E). After such training, transitive inference is tested by presenting subjects with the non-adjacent stimuli B and D (von Fersen et al., 1991; Delius and Siemann, 1998; Acuna et al., 2002). If the bees established a hierarchical order of stimuli (i.e., A > B > C > D > E), they would be expected to prefer B over D. If no preference is observed, it suggests that choices were guided by the associative strength of the stimuli, as B and D have an identical reinforcement history.

Our study employed the Pavlovian protocol of olfactory conditioning of the proboscis extension response (PER) (Bitterman et al., 1983; Giurfa and Sandoz, 2012), allowing for full control of reinforcement delivery—a control not achieved in previous studies on transitive inference in bees and wasps that used operant setups (Benard and Giurfa, 2004; Tibbetts et al., 2019). Across three experiments, we established a stimulus hierarchy based on varied training sequences; however, when it came to the comparison between B and D, the bees did not show a preference for the stimulus higher in the hierarchy over the lower one. This lack of preference indicates that their choices were primarily driven by the associative strength of the stimuli, as both B and D were equally reinforced (forward-paired with sucrose solution) and non-reinforced (backward-paired with sucrose solution).

### Honey bee performance in operant vs. Pavlovian conditioning regimes for transitive inferences

The present results align with findings from a prior study on transitive inferences in honey bees, which used an operant approach (Benard and Giurfa, 2004). In that study, free-flying bees were trained to fly into a Y-maze to discriminate between five distinct black and white patterns, some associated with a sucrose reward and others not. The patterns were arranged into four overlapping premise pairs (A+ vs. B–, B+ vs. C–, C+ vs. D–, D+ vs. E–, with “+” indicating sucrose presence and “–” indicating absence). Bees were then tested in the maze with non-adjacent pairs A vs. E and B vs. D. Consistent with our findings, bees preferred A over E but showed no preference between B and D, indicating that their choices were guided by the associative strength of each stimulus rather than by an implicit stimulus hierarchy based on transitive inferences. Additionally, a recency effect was observed, where the most recently rewarded stimulus influenced their choices more strongly (Benard and Giurfa, 2004).

The study on free-flying bees shared with ours the use of an appetitive context, as sucrose solution was used as a reward for correct responses in both cases. However, the two studies differ in that the former trained freely moving bees with visual stimuli, whereas our work involved harnessed bees conditioned with olfactory stimuli. The key distinction lies in the use of an operant context in the previous study and a Pavlovian context in the present one. The Pavlovian approach, unlike the operant setup, allows precise control over reinforced and non-reinforced choices.

In both contexts, the conditioning schedule assumes that B and D have equivalent associative strengths since they are equally reinforced and non-reinforced. However, in the operant paradigm, B and D may not be truly equivalent, as the operant setup does not strictly ensure equal exposure to each stimulus and its associated renforcer. For instance, since stimulus A at the top of the hierarchy is always rewarded, the adjacent stimulus B might be chosen and penalized less frequently. Conversely, the stimulus E at the bottom, which is consistently unrewarded, may lead to more frequent choices of its rewarded counterpart, D. Consequently, B could indirectly gain associative strength from proximity to A, while D might lose associative strength from proximity to E. This could lead to a preference for B over D, not due to transitive inference but rather based on differences in associative strength (Delius and Siemann, 1998). Although this issue was not apparent in the free-flying bee experiments, where no B-over-D preference emerged (Benard and Giurfa, 2004), it underscores the importance of fully controlling an animal’s experience in transitive-inference studies to conclude about its capacity to build implicit stimulus hierarchies.

### An associative perspective on honey bee performance following transitive-inference training

Algebraic models have been proposed to explain how transitive inferences are constructed without relying on deductive reasoning or a hierarchical ranking of stimuli (von Fersen et al., 1991; Couvillon and Bitterman, 1992; Wynne, 1997; Siemann and Delius, 1998). In these conditioning models, the associative value of a stimulus increases if its selection is reinforced and decreases if it is not. These models do not assume higher-order cognitive processes; instead, they account for transitive performance based on the associative strength acquired by each stimulus during training with the stimulus pairs. According to this view, sequential training endows each stimulus with associative values that happen to reflect an order similar to what deductive reasoning would infer (i.e., A with the highest associative value, followed by B, down to E with the lowest associative value). Thus, when presented with the novel pair B vs. D, a subject’s preference for B over D may simply result from selecting the stimulus with the higher associative value. While these models have successfully predicted performance in various transitive inference experiments across different species (Vasconcelos, 2008), they have failed in some instances where reinforcement history showed no strong influence on transitive choices (Weaver et al., 1997; Lazareva and Wasserman, 2006, 2012; Jensen et al., 2017).

In our experiments, the lack of preference between B and D made the associative explanation of the bees’ performance straightforward. It also indicates that the phenomenon known as “value transfer” —the idea that during the training of an X + Y- discrimination, some of the associative strength acquired by X+ transfers to Y- (von Fersen et al., 1991; Zentall and Sherburne, 1994; Zentall et al., 1996a; Zentall et al., 1996b) —did not influence the bees’ performance. In our experiments, the response levels for B and D were identical across all cases. If value transfer had occurred, bees would be expected to show a preference for the stimulus closer to the one that was always rewarded (e.g., preferring B over D).

In all cases, the bees significantly preferred the stimulus at the top of the hierarchy (i.e., the stimulus always rewarded, either A or E, depending on the experiment) and responded significantly less to the stimulus at the bottom of the hierarchy (i.e., the stimulus never rewarded, either E or A, depending on the experiment). Overall, these findings indicate that the reinforcement history of each stimulus was the key factor guiding the bees’ responses.

### The recency effect and its influence on honey bee choices

The recency effect is a cognitive bias in which individuals place greater emphasis on the most recent events, often impacting their decision-making (Baddeley and Hitch, 1993). In addition to relying on the associative strength of odorants, bee choices were also partially influenced by this recency effect. Specifically, the reinforcement assigned to odorants in the final conditioning phase biased their choices, leading to a decrease or increase in responses during the final tests.

In Experiment 1, the last conditioning pair experienced by *Group I* and *Group II* was D+ vs. –E, and –D+ vs. E+, respectively. In both cases, the recency effect worked in conjunction with associative strength to produce the lowest response level to E in *Grou*p I—where E was never rewarded throughout training—and the highest response level to E in *Group II*—where E was always rewarded. In *Group III* and *Group IV,* the recency effect became more apparent as the training sequences did not include experiences with the non-ambiguous stimuli (i.e., always reinforced or always non-reinforced) at the beginning and end of training. The last conditioning pairs experienced by *Groups III* and *Group IV* were B+ vs. –C and –B+ vs. C+, respectively. Consequently, in the final tests, *Group III* bees showed a decrease in responses to C while *Group IV* bees exhibited the opposite trend. Finally, for *Group V,* the final conditioning pair was C+ vs.–D, resulting in an increased preference for C in the final tests. This increase can be attributed to a recency effect, as C was not only the last rewarded odorant but also a ‘less ambiguous’ stimulus than D, given that the training pair in which –D was experienced as D+ occurred closer to the last conditioning phase than the pair where C+ was experienced as –C (see Table 1). Thus, a consistent recency effect was observed in the bees’ performance across all experiments.

### Wasps and bees: a real difference in solving transitive inference?

Unlike honey bees, wasps have been reported to solve transitive inference tasks by creating a hierarchical ranking of stimuli experienced during training (Tibbetts et al., 2019). In this study, two species of *Polistes* wasps (*P. dominula* and *P. metricus*) were trained with a transitive-inference schedule in an aversive context. The wasps were placed in a rectangular box with each end displaying a different color, where one color was paired with an electric shock, and the other was safe. Five colors were used in total, arranged into four overlapping premise pairs (A– vs. B+, B– vs. C+, C– vs. D+, D– vs. E+, where + and – indicate the presence or absence of an electric shock, respectively). When tested with non-adjacent pairs A (never shocked) vs. E (always shocked) and B vs. D, the wasps showed a preference for A over E, and for B over D, indicating that D was ranked higher in the aversive hierarchy. These results were presented as the first demonstration of transitive-inference judgments in an invertebrate (Tibbetts et al., 2019).

On one hand, it could be argued that wasps, unlike bees, were able to establish a hierarchy of stimuli on an aversive scale through transitive inference because their biology has equipped them for such tasks. The social structure of wasps depends on well-defined individual hierarchies, where conflicts can lead to aversive outcomes (Jandt et al., 2013). This natural context may help explain their success in solving transitive inference tasks, especially when negative reinforcement is applied for incorrect choices (Tibbetts et al., 2019).

However, caution is warranted before drawing conclusions, particularly given the operant context of these experiments and the lack of precise quantification of wasp behavior during training. In the study with free-flying honey bees (Benard and Giurfa, 2004), reinforced and non-reinforced experiences were quantified during training, while the bees were freely choosing within a maze. To avoid concluding about transitive inference without precise control of reinforcement history, the reward/penalty ratios (R) of stimuli B and D were calculated based on the bees’ choices during training. The percentage of choices when the stimulus was rewarded (correct choices) was divided by the percentage of choices when it was non-rewarded (incorrect choices). Interestingly, and consistent with the idea that bees might distribute their actions differently between B and D in response to the operant context, R_B_ was always greater than R_D_. Therefore, had bees in these experiments preferred B over D (which they did not), attributing their choices to transitive inference would have been incorrect, as an associative explanation based on differing R values for B and D could account for the behavior observed.

The same argument applies to the wasp experiment, where, in contrast to the bee study, a preference for B (the safest stimulus) over D was observed. This work did not quantify the time spent at stimuli B and D in safe and punished trials during training, which could have revealed differences in exposure to aversive and appetitive (safe) reinforcing situations. Thus, caution is necessary before concluding that wasps organized the trained stimuli into an implicit hierarchy, as alternative explanations have not been ruled out.

### Honey bee failure in the transitive-inference task: a cognitive limit

Does honey bees’ inability to establish an implicit stimulus hierarchy from a transitive-inference task suggest they rely exclusively on reinforcement history to guide foraging decisions? Associative models of bee foraging and decision-making have been developed (Greggers and Menzel, 1993; Montague et al., 1995), drawing on frameworks like the Rescorla-Wagner model (Rescorla and Wagner, 1972). While these models account for bees’ decisions in artificial flower patch setups, bees are not simply associative machines. They exhibit remarkable cognitive abilities that go beyond basic associative learning (Menzel and Giurfa, 2001; Giurfa, 2003, 2007; Avarguès-Weber and Giurfa, 2014). For instance, bees can learn to categorize unfamiliar objects based on shared visual characteristics (van Hateren et al., 1990; Giurfa et al., 1996; Stach et al., 2004; Benard et al., 2006), and perform discriminations based on abstract concepts like “sameness” (Giurfa et al., 2001), spatial relationships (Avarguès-Weber et al., 2011; Avargues-Weber et al., 2012), and numerosity, including concepts of zero and a mental number line (Gross et al., 2009; Pahl et al., 2013; Howard et al., 2018; Giurfa, 2019a, 2019b; Howard et al., 2019; Giurfa et al., 2022). These examples of higher-order learning suggest that pure associative models cannot fully explain bees’ cognitive achievements (Giurfa, 2015).

Given these impressive cognitive capacities, why, then, do bees appear unable to establish a hierarchical ordering of odors experienced during training? One explanation might be that this task exceeds their cognitive abilities, possibly due to limitations in their memory organization. Honey bees exhibit a behavior known as “flower constancy,” meaning they temporarily specialize in a single flower species as long as it remains productive (Grant, 1950; Free, 1963; Waser, 1986; Chittka et al., 1999). This constancy relies on bees’ ability to memorize the characteristics of the flowers they are currently exploiting (Menzel, 1985, 1999). Long-term memories can form after only a few trials and are stabilized through protein synthesis (Wüstenberg et al., 1998). In some cases, precise control of appetitive motivation enables the formation of these memories after a single learning trial (Villar et al., 2020). However, these appetitive memories are specific to the flower species being exploited, guiding foraging behavior until the memorized information is replaced or extinguished when new flowers are available (Menzel, 1985, 1999). Bees do not typically forage on multiple species simultaneously, so they do not establish parallel, concurrent appetitive memories. Thus, inferring a stimulus hierarchy through a transitive-inference task may exceed bees’ cognitive limits, as it requires storing and comparing multiple appetitive memories—three of which (B, C, D) — involve reinforcement reversals. Whether this argument applies to aversive memories remains to be determined by training bees in a transitive-inference design within an aversive context, where they learn to associate odorants with electric shocks (Vergoz et al., 2007; Giurfa et al., 2009; Roussel et al., 2009).

Notably, two studies (Benard and Giurfa, 2004 and the present study) reached similar results and conclusions despite different experimental designs and behavioral contexts—operant learning of visual stimuli in free-flying bees and Pavlovian olfactory learning in restrained bees. This consistency suggests that bees’ failure to form an implicit stimulus hierarchy after transitive-inference training may indeed reflect a cognitive limitation. This finding is important in comparative cognition studies, where it is essential not only to identify what animals can achieve but also to understand their limitations. We hope our results inspire further research on honey bees’ cognitive complexity and its inherent boundaries.

## Data Availability

The datasets presented in this study can be found in online repositories. The names of the repository/repositories and accession number(s) can be found at: https://doi.org/10.6084/m9.figshare.27798498.
