# Supplementary material for: Honey bees rely on associative stimulus strength after training on an olfactory transitive inference task
Source: Front Psychol. 2025 Jan 7;15:1529460. doi: 10.3389/fpsyg.2024.1529460 (PMC11747915; doi:10.3389/fpsyg.2024.1529460)
Supplement: Supplementary file 1 [file Data_Sheet_1.PDF]

Table S1 : GLMER effects summary and post hoc tables for Fig. 1A

Model selected → Model 1: PER ~ Trial*Phase +(1|BeeID)

	Main and interaction effects predicting PER responses in Fig. 1A			
Fixed effects	Estimate	SE	z	p-value
Phase B+	14.77	16.41	0.90	0.368
Phase C+	16.28	16.40	0.99	0.321
Phase D+	15.90	16.40	0.97	0.333
Trial2	15.68	16.41	0.96	0.271
Trial3	18.20	16.40	1.11	0.339
Trial4	20.20	16.40	1.23	0.267
Trial5	21.00	16.41	1.28	0.218
Trial6	21.00	16.40	1.28	0.210

Calculated using the Likelihood ratio test (LRT) to assess the significance of the interaction as a whole

	$\chi 2$	df	p-value
Trial*Phase	43.86	15	0.01 ***

	Post-hoc analysis for Phase:Trial interaction			
Trial 1				
Contrast	Estimate	SE	z	p-value
(A+) vs. (B+)	-14.8	16.40	-0.90	0.80
(A+) vs. (C+)	-16.3	16.40	-0.99	0.75
(A+) vs. (D+)	-15.9	16.40	-0.97	0.76
(B+) vs. (C+)	-1.51	0.77	-1.96	0.20
(B+) vs. (D+)	-1.12	0.79	-1.42	0.49
(C+) vs. (D+)	0.38	0.62	0.62	0.93
Trial 2				
Contrast	Estimate	SE	z	p-value
(A+) vs. (B+)	0.91	0.80	1.12	0.67
(A+) vs. (C+)	-0.93	0.63	-1.49	0.44
(A+) vs. (D+)	-1.09	0.62	-1.75	0.30
(B+) vs. (C+)	-1.84	0.76	-2.43	0.07 .
(B+) vs. (D+)	-2.00	0.75	-2.65	0.04 *
(C+) vs. (D+)	0.15	0.55	-0.27	0.99
Trial 3				
Contrast	Estimate	SE	z	p-value
(A+) vs. (B+)	1.29	0.52	2.49	0.06 .
(A+) vs. (C+)	0.63	0.50	1.25	0.60
(A+) vs. (D+)	0.63	0.50	1.25	0.60
(B+) vs. (C+)	-0.66	0.52	-1.29	0.57
(B+) vs. (D+)	-0.66	0.52	-1.29	0.57
(C+) vs. (D+)	0.00	0.50	0.003	1.00
Trial 4				
Contrast	Estimate	SE	z	p-value
(A+) vs. (B+)	2.13	0.59	3.59	0.002 **
(A+) vs. (C+)	0.58	0.63	0.93	0.79
(A+) vs. (D+)	1.62	0.60	2.71	0.03 *

(B+) vs. (C+)	-1.55	0.55	-2.82	0.02 *
(B+) vs. (D+)	-0.51	0.51	-1.01	0.74
(C+) vs. (D+)	1.03	0.55	1.88	0.24

Trial 5				
Contrast	Estimate	SE	z	p-value
(A+) vs. (B+)	1.70	0.70	2.42	0.07 .
(A+) vs. (C+)	1.38	0.71	1.93	0.21
(A+) vs. (D+)	1.86	0.70	2.66	0.04 *
(B+) vs. (C+)	-0.33	0.57	-0.57	0.94
(B+) vs. (D+)	0.15	0.55	0.28	0.99
(C+) vs. (D+)	0.48	0.57	0.85	0.83

Trial 6				
Contrast	Estimate	SE	z	p-value
(A+) vs. (B+)	1.70	0.70	2.43	0.07 .
(A+) vs. (C+)	1.55	0.71	2.18	0.13
(A+) vs. (D+)	1.54	0.71	2.18	0.13
(B+) vs. (C+)	-0.16	0.56	-0.28	0.99
(B+) vs. (D+)	-0.16	0.56	-0.28	0.99
(C+) vs. (D+)	-0.008	0.57	-0.001	1.00

Table S2 : GLMER effects summary and post hoc tables for Fig. 1D

Model selected → Model 2: PER ~ Trial + Phase +(1|BeeID)

	Main effects predicting PER responses in Fig. 1D			
Fixed effects	Estimate	SE	z	p-value
Phase C+	1.09	0.21	5.25	1.51e-7 ***
Phase D+	0.53	0.20	2.60	0.009 **
Phase E+	1.55	0.21	7.08	1.46e-12 ***
Trial2	0.91	0.24	3.81	0.0001 ***
Trial3	1.32	0.24	5.48	4.38e-8 ***
Trial4	2.24	0.26	8.70	< 2e-16 ***
Trial5	2.73	0.28	9.94	< 2e-16 ***
Trial6	3.36	0.31	10.83	< 2e-16 ***

	Post-hoc analysis for Phase effect			
Contrast	Estimate	SE	z	p-value
(B+) vs. (C+)	-1.10	0.21	-5.25	<0.0001 ***
(B+) vs. (D+)	-0.53	0.20	-2.60	0.046 *
(B+) vs. (E+)	-15.9	0.22	-7.08	<0.0001 ***
(C+) vs. (D+)	-1.51	0.21	2.76	0.029 *
(C+) vs. (E+)	-1.12	0.22	-2.05	0.168
(D+) vs. (E+)	0.38	0.22	-4.73	<0.0001 ***

	Post-hoc analysis for Trial effect			
Contrast	Estimate	SE	z	p-value
T1 vs. T2	-0.91	0.24	-3.81	0.002 **

T1 vs. T3	-1.32	0.24	-5.48	<0.0001 ***
T1 vs. T4	-2.24	0.26	-8.70	<0.0001 ***
T1 vs. T5	-2.73	0.28	-9.94	<0.0001 ***
T1 vs. T6	-3.36	0.31	-10.83	<0.0001 ***
T2 vs. T3	-0.41	0.23	-1.80	0.47
T2 vs. T4	-1.32	0.24	-5.48	<0.0001 ***
T2 vs. T5	-1.82	0.26	-7.01	<0.0001 ***
T2 vs. T6	-2.44	0.30	-8.27	<0.0001 ***
T3 vs. T4	-0.92	0.24	-3.81	0.002 **
T3 vs. T5	-1.41	0.26	-5.47	<0.0001 ***
T3 vs. T6	-2.04	0.29	-6.93	<0.0001 ***
T4 vs. T5	-0.50	0.27	-1.85	0.43
T4 vs. T6	-1.12	0.30	-3.71	0.003 **
T5 vs. T6	-0.63	0.32	-1.98	0.35

Table S3 : GLMER effects summary and post hoc tables for Fig. 2A

Model selected → Model 2: PER ~ Trial + Phase +(1|BeeID)

	Main and interaction effects predicting PER responses in Fig. 2A			
Fixed effects	Estimate	SE	z	p-value
Phase A+	-0.32	0.24	-1.33	0.18
Phase B+	-2.58	0.28	-9.28	< 2e-16 ***
Phase D+	-2.41	0.27	-8.83	< 2e-16 ***
Trial2	1.37	0.44	1.93	0.002 **
Trial3	2.92	0.43	4.10	7.49e-12 ***
Trial4	3.69	0.43	5.11	< 2e-16 ***
Trial5	4.45	0.45	5.49	< 2e-16 ***
Trial6	4.69	0.45	4.94	< 2e-16 ***

	Post-hoc analysis for Phase effect			
Contrast	Estimate	SE	z	p-value
(C+) vs. (A+)	0.32	0.24	1.33	0.54
(C+) vs. (B+)	2.58	0.28	9.28	<0.0001 ***
(C+) vs. (D+)	2.41	0.27	8.83	<0.0001 ***
(A+) vs. (B+)	2.26	0.27	8.34	<0.0001 ***
(A+) vs. (D+)	2.09	0.27	7.86	<0.0001 ***
(B+) vs. (D+)	-0.17	0.26	-0.65	0.18

	Post-hoc analysis for Trial effect			
Contrast	Estimate	SE	z	p-value
T1 vs. T2	-1.37	0.44	-3.09	0.02 *
T1 vs. T3	-2.92	0.43	-6.85	<0.0001 ***
T1 vs. T4	-3.69	0.43	-8.52	<0.0001 ***
T1 vs. T5	-4.45	0.45	-9.99	<0.0001 ***
T1 vs. T6	-4.69	0.45	-10.40	<0.0001 ***
T2 vs. T3	-1.56	0.32	-4.82	<0.0001 ***
T2 vs. T4	-2.32	0.33	-7.09	<0.0001 ***
T2 vs. T5	-3.08	0.34	-9.04	<0.0001 ***

T2 vs. T6	-3.32	0.35	-9.58	<0.0001 ***
T3 vs. T4	-0.77	0.28	-2.74	0.07
T3 vs. T5	-1.53	0.29	-5.29	<0.0001 ***
T3 vs. T6	-1.76	0.29	-6.02	<0.0001 ***
T4 vs. T5	-0.76	0.28	-2.73	0.07
T4 vs. T6	-0.99	0.28	-3.54	0.005 **
T5 vs. T6	-0.24	0.28	-0.84	0.96

Table S4 : GLMER effects summary and post hoc tables for Fig. 2D

Model selected → Model 2: PER ~ Trial + Phase +(1|BeeID)

	Main effects predicting PER responses in Fig. 2D			
Fixed effects	Estimate	SE	z	p-value
Phase B+	0.42	0.22	1.89	0.059 .
Phase C+	-1.19	0.22	-5.28	1.26e-7 ***
Phase E+	-0.17	0.22	0.77	0.44
Trial2	0.76	0.60	2.70	0.007 **
Trial3	1.77	0.59	6.40	1.59e-10 ***
Trial4	2.58	0.62	8.93	< 2e-16 ***
Trial5	3.39	0.63	10.76	< 2e-16 ***
Trial6	3.13	0.61	10.27	< 2e-16 ***

	Post-hoc analysis for Phase effect			
Contrast	Estimate	SE	z	p-value
(D+) vs. (B+)	-0.42	0.22	-1.89	0.23
(D+) vs. (C+)	1.19	0.22	5.29	<0.0001 ***
(D+) vs. (E+)	-0.17	0.22	-0.77	0.87
(B+) vs. (C+)	1.60	0.23	6.96	<0.0001 ***
(B+) vs. (E+)	0.25	0.22	1.12	0.68
(C+) vs. (E+)	-1.36	0.23	-5.98	<0.0001 ***

	Post-hoc analysis for Trial effect			
Contrast	Estimate	SE	z	p-value
T1 vs. T2	-0.76	0.28	-2.70	0.07 .
T1 vs. T3	-1.77	0.28	-6.40	<0.0001 ***
T1 vs. T4	-2.58	0.29	-8.93	<0.0001 ***
T1 vs. T5	-3.39	0.32	-10.76	<0.0001 ***
T1 vs. T6	-3.13	0.31	-10.27	<0.0001 ***
T2 vs. T3	-1.01	0.25	-4.01	0.0009 **
T2 vs. T4	-1.83	0.27	-6.90	<0.0001 ***
T2 vs. T5	-2.63	0.29	-9.04	<0.0001 ***
T2 vs. T6	-2.38	0.28	-8.45	<0.0001 ***
T3 vs. T4	-0.81	0.25	-3.21	0.017 *
T3 vs. T5	-1.62	0.28	-5.82	<0.0001 ***
T3 vs. T6	-1.36	0.27	-5.08	<0.0001 ***
T4 vs. T5	-0.81	0.28	-2.87	0.048 *
T4 vs. T6	-0.55	0.27	-2.02	0.33

T5 vs. T6	-0.26	0.29	0.88	0.95

Table S5 : GLMER effects summary and post hoc tables for Fig. 3A

Model selected → Model 2: PER ~ Trial + Phase +(1|BeeID)

	Main effects predicting PER responses in Fig. 3A			
Fixed effects	Estimate	SE	z	p-value
Phase A+	0.32	0.22	1.47	0.14
Phase B+	-1.06	0.25	-4.29	1.82e-5 ***
Phase D+	-1.21	0.25	-4.77	1.81e-6 ***
Trial2	3.37	0.77	4.39	1.12e-5 ***
Trial3	4.16	0.76	5.44	5.30e-8 ***
Trial4	4.52	0.76	5.92	3.19e-9 ***
Trial5	4.38	0.76	5.74	9.78e-9 ***
Trial6	4.86	0.76	6.36	2.01e-10 ***

	Post-hoc analysis for Phase effect			
Contrast	Estimate	SE	z	p-value
(C+) vs. (A+)	-0.33	0.22	-1.47	0.45
(C+) vs. (B+)	1.06	0.25	4.29	0.0001 ***
(C+) vs. (D+)	1.21	0.25	4.77	<0.0001 ***
(A+) vs. (B+)	1.39	0.25	5.61	<0.0001 ***
(A+) vs. (D+)	1.54	0.25	6.07	<0.0001 ***
(B+) vs. (D+)	0.15	0.27	0.54	0.95

	Post-hoc analysis for Trial effect			
Contrast	Estimate	SE	z	p-value
T1 vs. T2	-3.37	0.77	-4.40	0.0002 **
T1 vs. T3	-4.16	0.76	-5.44	<0.0001 ***
T1 vs. T4	-4.52	0.76	-5.92	<0.0001 ***
T1 vs. T5	-4.38	0.76	-5.74	<0.0001 ***
T1 vs. T6	-4.86	0.75	-6.36	<0.0001 ***
T2 vs. T3	-0.78	0.30	-2.64	0.09
T2 vs. T4	-1.15	0.29	-3.92	0.0012 **
T2 vs. T5	-1.01	0.29	-3.42	0.0082 **
T2 vs. T6	-1.49	0.29	-5.10	<0.0001 ***
T3 vs. T4	-0.37	0.27	-1.36	0.75
T3 vs. T5	-0.22	0.27	-0.83	0.96
T3 vs. T6	-0.71	0.27	-2.66	0.08
T4 vs. T5	0.14	0.27	0.54	0.99
T4 vs. T6	-0.34	0.26	-1.31	0.77
T5 vs. T6	-0.48	0.26	-1.85	0.43
